# Inhaled Gases as Therapies for Post–Cardiac Arrest Syndrome: A Narrative Review of Recent Developments

**DOI:** 10.3389/fmed.2020.586229

**Published:** 2021-01-14

**Authors:** Kei Hayashida, Santiago J. Miyara, Koichiro Shinozaki, Ryosuke Takegawa, Tai Yin, Daniel M. Rolston, Rishabh C. Choudhary, Sara Guevara, Ernesto P. Molmenti, Lance B. Becker

**Affiliations:** ^1^Laboratory for Critical Care Physiology, Feinstein Institutes for Medical Research, Northwell Health System, Manhasset, NY, United States; ^2^Department of Emergency Medicine, North Shore University Hospital, Northwell Health System, Manhasset, NY, United States; ^3^Elmezzi Graduate School of Molecular Medicine, Manhasset, NY, United States; ^4^Department of Surgery, Medicine, and Pediatrics, Zucker School of Medicine at Hofstra/Northwell, New York, NY, United States; ^5^Institute of Health Innovations and Outcomes Research, Feinstein Institutes for Medical Research, Northwell Health, Manhasset, NY, United States; ^6^Department of Surgery, Northwell Health, Manhasset, NY, United States; ^7^Donald and Barbara Zucker School of Medicine at Hofstra/Northwell, Northwell Health, Hempstead, NY, United States

**Keywords:** cardiac arrest, cardiopulmonary resuscitation, ischemia-reperfusion injury, neuroprotection, nitric oxide, xenon, molecular hydrogen (H_2_), PCAS

## Abstract

Despite recent advances in the management of post–cardiac arrest syndrome (PCAS), the survival rate, without neurologic sequelae after resuscitation, remains very low. Whole-body ischemia, followed by reperfusion after cardiac arrest (CA), contributes to PCAS, for which established pharmaceutical interventions are still lacking. It has been shown that a number of different processes can ultimately lead to neuronal injury and cell death in the pathology of PCAS, including vasoconstriction, protein modification, impaired mitochondrial respiration, cell death signaling, inflammation, and excessive oxidative stress. Recently, the pathophysiological effects of inhaled gases including nitric oxide (NO), molecular hydrogen (H_2_), and xenon (Xe) have attracted much attention. Herein, we summarize recent literature on the application of NO, H_2_, and Xe for treating PCAS. Recent basic and clinical research has shown that these gases have cytoprotective effects against PCAS. Nevertheless, there are likely differences in the mechanisms by which these gases modulate reperfusion injury after CA. Further preclinical and clinical studies examining the combinations of standard post-CA care and inhaled gas treatment to prevent ischemia–reperfusion injury are warranted to improve outcomes in patients who are being failed by our current therapies.

## Introduction

Cardiac arrest (CA) is a significant cause of death worldwide; ~356,000 cases occur out-of-hospital (OHCA) (1), and 200,000 cases occur in-hospital (IHCA) (2) per year in the United States. In recent years, rates of layperson-initiated cardiopulmonary resuscitation (CPR) and layperson use of automated external defibrillators have increased over time (1). Despite such recent advances in social awareness and management of CA, the survival rate without neurologic sequelae after resuscitation remains very low, representing a public health challenge (1–5). To date, no specific pharmaceutical drugs are effective against post-CA syndrome (PCAS) (3–5).

Over the past decades, resuscitation guidelines have emphasized the lifesaving value of high-quality CPR methods and rapid defibrillation for achieving the return of spontaneous circulation (ROSC), as well as treatment strategies such as hypothermia for post-CA brain injury. In 2002, two randomized controlled trials (RCTs) showed that therapeutic hypothermia (TH) significantly improved long-term outcomes in patients with PCAS who presented with ventricular fibrillation (VF) as an initial rhythm, drawing attention to the multidisciplinary treatment approach for those patients (3, 4, 6, 7). However, a large European RCT conducted by Nielsen et al. in 2013, including 939 comatose patients after ROSC, showed no significant benefit of improving the neurological outcomes when TH cooled down to 33°C compared to the management at a near-normal temperature of 36°C (8). This report questioned the effectiveness of TH for PCAS, which has been recommended in the international guidelines for a decade. In light of this controversy, a large RCT conducted by Bernard et al. in 2016, including 1,198 OHCA patients, demonstrated that the induction of mild TH, using a rapid large-volume intravenous cold saline infusion during CPR, indeed decreased the rate of ROSC in adult patients with an initial shockable rhythm and did not improve the survival rate (9). Recent RCT conducted by Lascarrou et al. in 2019 has evaluated targeted temperature management (TTM) for comatose patients who had been resuscitated from CA with non-shockable rhythm. They concluded that moderate TH at 33°C for 24 h led to a higher survival with a favorable neurologic outcome at 90 days compared to targeted normothermia (10). Also, the latest American Heart Association guidelines recommended TTM for comatose adults after ROSC from OHCA and IHCA with any initial rhythm (11). Despite these evidences, many uncertainties within the topic of TTM remain, and therefore, the development of alternative approaches with or without TTM is an unmet medical need in improving the prognosis of PCAS.

Prolonged ischemia during CA results in a variety of cellular insults. After achieving ROSC, ischemia–reperfusion injury (IRI) causes oxidative stress in the reperfused tissues, leading to exacerbation of the cellular injury (12). Recently, it has been shown in several scientific publications that nitric oxide (NO) (13), molecular hydrogen (H_2_) (14), xenon (Xe) (15), carbon monoxide (16), argon (17), and hydrogen sulfide (18) have protective effects against organ injuries related to IRI. In general, gases are small molecules; therefore, they have excellent diffusivity and easily permeate the cell membrane, targeting different organelles including the mitochondria and the nuclei. Especially, the cytoprotective effects of NO, H_2_, and Xe have attracted much attention in PCAS in not only animal models but also clinical settings. Therefore, the scope of this review is to describe those selected gases that have transitioned from bench to bedside and that have been already administered in patients. Herein, we briefly introduce the pathophysiology of PCAS and present a review of recent biomedical research developments on NO, H_2_, and Xe that have been proposed in recent literature.

## Post-CA Syndrome

PCAS is described as a unique and complex pathophysiological condition that involves (a) systemic IRI, (b) post-CA brain injury, and (c) post-CA myocardial dysfunction (3, 4). This condition is often complicated by a fourth component: the unsolved condition that caused the CA (3).

All clinical and biological manifestations associated with PCAS are putatively attributed to the IRI in vital organs including the brain and heart (3–5). The whole-body IRI with consequent oxygen debt causes a generalized activation of the cell-mediated immunologic response, vascular endothelial damage, hypercoagulability, and immunosuppression (3, 19–21). It has been observed that sharp increases in various cytokines occur in the bloodstream as early as 3 h after CA. Several cytokines have shown greater elevations in non-survivors than in survivors (20). Accordingly, it has been proposed that the pathophysiology of PCAS has several similar features as those of sepsis (19). The causes of post-CA organ damage may include increased activation of leukocytes, upregulated cytokines production, intracellular Ca^2+^ overload, mitochondrial dysfunction (22), and the generation of excessive reactive oxygen species (ROS) (23, 24). Excessive ROS production leads to DNA damage and lipid peroxidation, ultimately resulting in increased necrosis, apoptosis, and necroptosis (12, 25, 26). Compelling evidence has shown that mitochondria play a crucial role as effectors and targets of IRI (27–32). In fact, mitochondria are considered as one of the most susceptible subcellular targets of brain ischemia (33–35). A dysfunctional mitochondrial electron transport chain (METC) can result in an electron “leakage” phenomenon, reduced free oxygen, and the utilization of oxygen as an ubiquitous electron donor (substrate) to produce ROS (36). A body of evidence from preclinical studies has demonstrated that post-CA normoxic therapy improves neurological impairment, histological neuronal cell death, and cerebral metabolism (37–42).

Post-CA brain injury includes anoxic neuronal degeneration due to global ischemia during CA and/or shortly after ROSC, as well as delayed neurodegeneration, which can ensue within hours or several days after CA (43, 44). In a cohort study of 187 patients who underwent brain autopsy after CA, histopathologically determined severe hypoxic–ischemic encephalopathy was observed in patients with bilaterally absent cortical somatosensory-evoked potentials, gray–white matter ratio of brain computed tomographic imaging <1.10, highly malignant electroencephalographic patterns, and serum neuron-specific enolase concentration > 67 μg/L (45). In response to the stress due to global ischemia, several cytokine/chemokines, adhesion molecules, and ROS are released by different cells, including leukocytes, endothelial cells, and activated platelets (46). Aberrant ROS generation causes damage to fatty acids in the cell membrane, leading to increased membrane permeability and disruption of the blood–brain barrier (BBB). Cell membrane damage and BBB disruption result in cell swelling and cerebral edema, which, in turn, leads to further exacerbation of brain ischemia. Hypoperfusion during CPR and/or shortly after ROSC leads to a mismatch between oxygen demand and supply, resulting in secondary hypoxia (47). The delayed neurodegeneration after ROSC involves complex and multiple mechanisms including cytotoxic free radical production, neuronal excitability, activation of apoptotic signaling pathways, intracellular Ca^2+^ overflow, and mitochondrial dysfunction, among others (22, 28, 30, 48). Neuronal cell damage in the brain regions that are vulnerable to ischemia, such as the hippocampus and cerebral cortex, becomes irreversible within a few hours after the onset of ischemia, thus requiring early therapeutic interventions. Notably, some evidence suggests that the brain function after ROSC could be preserved indirectly, supporting the homeostasis of damaged organs other than the brain itself (49).

Most cases of PCAS exhibit a widespread left ventricular wall motion abnormality that is transient and reversible, in cases of normal or near-normal coronary flow or non-cardiomyopathy. This phenomenon is called post-CA myocardial stunning, which has been recently recognized as a leading cause of early death after a successful ROSC (3). In one study assessing the prevalence of coronary artery disease and acute coronary artery occlusion after resuscitation for OHCA presenting with VF as an initial rhythm, significant coronary artery lesions were found in 71% (50). Approximately 30% of patients had significant coronary artery lesions even in the absence of chest pain symptoms before CA and ST-segment elevation after ROSC (50). A meta-analysis focusing on studies for OHCA patients pointed out that acute coronary angiography should be strongly considered irrespective of electrocardiographic findings, due to the high prevalence of coronary artery disease in patients without an obvious non-cardiac etiology (51). Preexisting coronary artery disease exacerbates the myocardial damage associated with PCAS. The presence of myocardium stunning prolongs the recovery of wall motion through IRI (52), which includes excessive ROS production (53) and Ca^2+^ overload (54, 55), resulting in hemodynamics destabilization after ROSC. In addition, clinical studies have shown that right ventricular or biventricular dysfunction can contribute to poor outcomes after ROSC (56, 57). Therefore, hemodynamic stabilization is particularly important to maintain adequate cerebral blood flow and prevent late-onset neuronal damage.

## Recent Developments in Gas Research as Therapeutic Agents For PCAS

In light of the limited clinical evidence supporting TH and other conventional approaches, recent preclinical studies have been focusing on alternative strategies that could increase neuroprotection immediately after ROSC. Significant attention has been paid to the possible use of inhaled gases such as NO, H_2_, and Xe, which have shown cytoprotective effects on organ injuries related to PCAS (13, 58–62). The main function of the lungs is to work as a gas exchanger, which allows oxygen to diffuse from the inhaled gas in the alveolus to the blood. The blood then carries and delivers oxygen to tissues to assist in the complex process of oxidative phosphorylation (63). Inhaled gas is a unique route of drug delivery, distinct from the intravenous or oral administration of medications, which allows for inhaled gaseous molecules to pass from the lung directly into the arterial circulatory system. Alternatively, it is conceivable that circulating cells are directly exposed to the gases as they pass through the pulmonary capillaries and may interact with or “pacified,” by a certain mechanism of each inhaled gas before the cells reach the reperfused peripheral tissues including the brain and heart.

### Nitric Oxide

**Figure 1:**
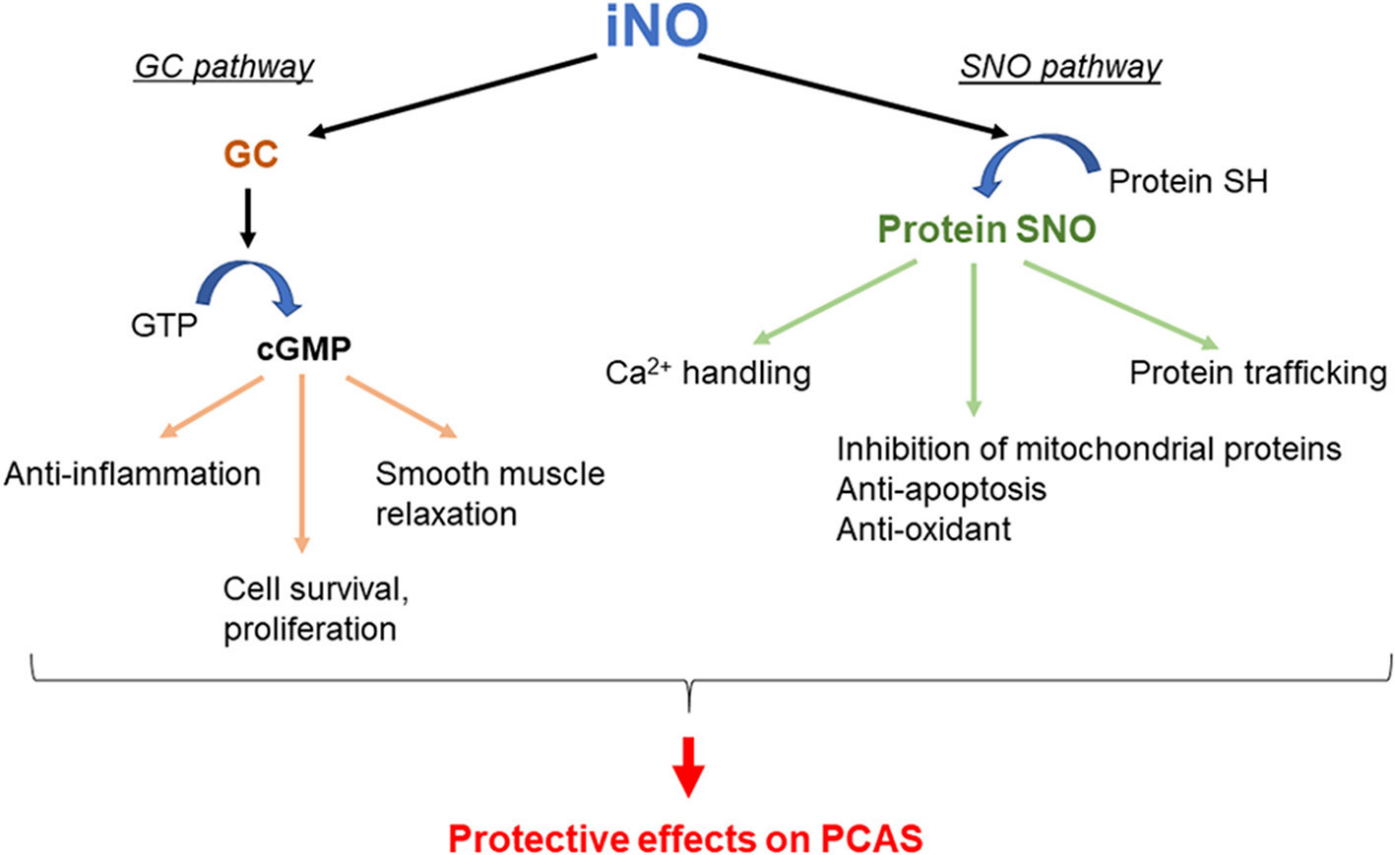
Potential mechanisms by which inhaled nitric oxide (iNO) improves outcomes in post–cardiac arrest syndrome (PCAS). GC, guanylyl cyclase; GTP, guanosine triphosphate; cGMP, cyclic guanosine monophosphate; SNO, S-nitrosylation.

The biological effects of NO are mediated through the activation of guanylyl cyclase (GC), followed by cyclic guanosine monophosphate (cGMP) production (GC pathway) (64). The biological effects of NO are also mediated through protein S-nitrosylation (SNO), which is the covalent attachment of NO to cysteine residues of target proteins (SNO pathway), by cGMP-independent mechanisms (65, 66). Both of these mechanisms have been implicated in the bioprotective effects of NO in IR disorders. Thus, several mechanisms that are responsible for the beneficial effects of NO on PCAS have been suggested (67). Potential mechanisms responsible for the beneficial effects of NO on the outcomes of PACS are shown in Figure 1. It has been reported that the administration of NO through inhalation (13, 58, 68, 69) or with an NO-donating compound (70) improves outcomes after CA in multiple species. Additionally, in mice lacking the NO synthase 3 gene, the protective effect of TH after CA/CPR is abolished (71), suggesting that NO may play an important role in TH. Furthermore, given the well-established pulmonary vasodilating effects of inhaled NO (72), it is conceivable that inhaled NO reduces the CA-induced pulmonary vascular resistance, thus enhancing the right-sided ventricular function and improving the outcomes of PCAS. Additionally, NO inhibits leukocyte adhesion (73) and migration (74), platelet activation (75), and acute inflammation (76). It has been reported that poor survival after CA/CPR is associated with leukocyte infiltration in the brain, heart, lung, liver, and kidney in mice (77, 78). It has also been demonstrated that NO is transported from the lung to the peripheral tissues through the hemoglobin, plasma protein SNOs, and nitrite ion generation and that NO in the periphery is released in the local ischemic region that exhibits acidosis where acid–base changes produce various physiological effects (79).

Minamishima et al. reported that NO breathing improves the outcomes after ROSC in mice by GC-dependent mechanisms (13). Wild-type mice were subjected to 7.5 min of potassium chloride–induced CA and subsequently resuscitated. One hour after CPR, mice were extubated and breathed air alone or air supplemented with 40 ppm NO for 23 h. The post-CA mice breathing air alone (air group) exhibited a poor 10-day survival rate (4 of 13 [30.7%]), depressed neurological and left ventricular function, increased caspase-3 activation, and cytokine driven inflammation in the brain. NO breathing attenuated the neurological and cardiac dysfunction 4 days after CA/CPR and markedly improved the 10-day survival rate (11 of 13 [84.6%]; *P* = 0.003 vs. air group) (13). They also found that GC-1α deletion abolished the ability of inhaled NO to inhibit the production of inflammatory cytokines in the brain and to improve the neurological function and survival rate after CA (13). These observations suggest that the protective effects of inhaled NO on outcomes after ROSC are largely mediated by GC-1α-dependent mechanisms. Another research group showed that NO inhalation starting at initiation of CPR until 30 min after ROSC prevented myocardial injury and improved neurologic function and survival in rats (68). It was also shown that NO breathing, starting with the left ventricular assist device–supported CPR for 5 h, increased the transpulmonary blood flow by reducing the pulmonary artery pressure and improving neurological outcomes in pigs (69). Moreover, inhaled NO improved pulmonary artery relaxation pressure during CPR, coronary perfusion pressure during the postresuscitation phase, and short-term survival in a porcine model of CA. Interestingly, these benefits occurred despite fewer vasopressor doses and shallower chest compressions (80).

On the other hand, the protein SNO pathway has recently attracted considerable attention (65, 66, 81). Protein SNOs have demonstrated the capacity to inhibit mitochondrial proteins such as complex I in the electron transport chain, cytochrome c oxidase, and F1F0ATPase (complex V), as well as to modulate mitochondrial ROS production, influence calcium-dependent opening of the mitochondrial permeability transition pore, promote selective importation of mitochondrial proteins, and stimulate mitochondrial fission (65, 81). Furthermore, SNO proteins play a crucial role in intracellular Ca^2+^ handling, protein trafficking, and regulation of cellular defense against apoptosis and oxidative stress (65).

**Figure 2:**
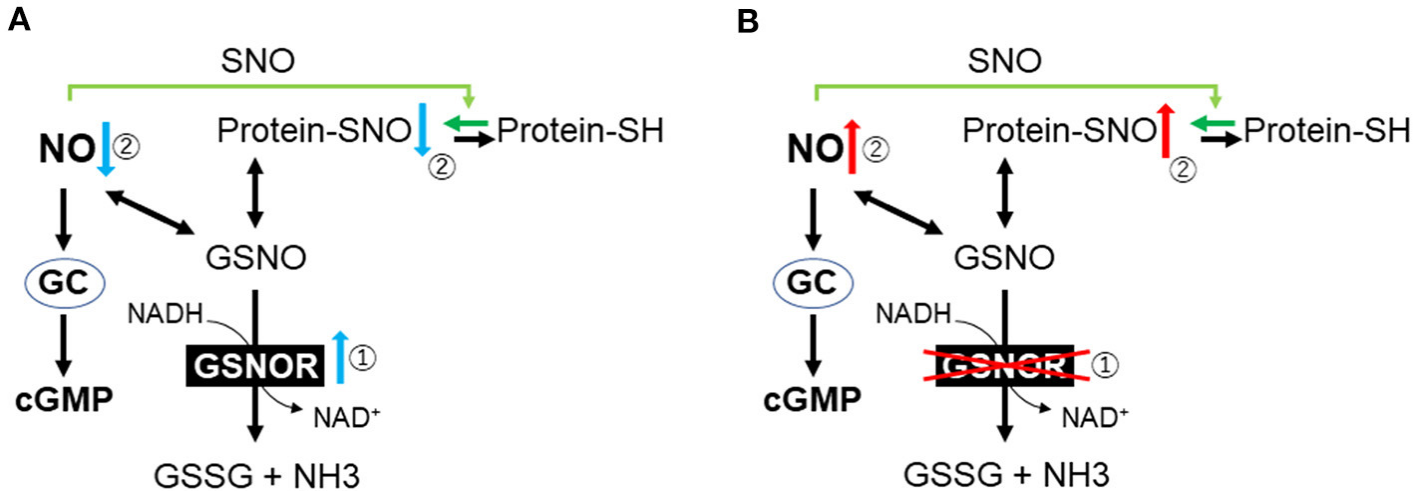
Outline of nitric oxide metabolism. **(A)** Cardiac arrest and resuscitation increase the activity of GSNOR. **(B)** Genetic or pharmacological inhibition of GSNOR increases the tissue levels of protein SNO and NO bioavailability. GC, guanylyl cyclase; cGMP, cyclic guanosine monophosphate; SH, cysteine thiols; GSNO, S-nitrosoglutathione; GSNOR, GSNO reductase; GSSG, glutathione disulfide; NH3, ammonia; NO, nitric oxide; SNO, S-nitrosylation.

S-nitrosoglutathione (GSNO), which is the most abundant intracellular S-nitrosothiol in human tissue, plays an important role as a reservoir of NO bioactivity (82). GSNO has potent antioxidant and anti-inflammatory effects in animal models of IR (83, 84). In physiological conditions, GSNO and protein SNOs remain at equilibrium, whereas GSNO reductase (GSNOR) centrally regulates the reduction of GSNO (Figure 2) (85). GSNOR is normally expressed in all tissues including the brain, liver, vascular endothelium, and smooth muscle cells (86). As GSNOR reduces the intracellular level of protein SNO and NO bioavailability, the genetic deletion or pharmacological inhibition of GSNOR has been reported to increase the tissue levels of the protein SNO, as well as to induce vasodilation and reduce inflammation. Previous animal studies suggest that GSNOR inhibition may be beneficial for systemic and brain inflammation as well as for ischemic cardiomyopathy (87–89).

To determine the role of GSNOR in the outcomes after CA/CPR, Hayashida et al. evaluated the effects of both GSNOR inhibitors and GSNOR gene deletion on the survival and neurological outcomes after CA in mice (90). They found that GSNOR activity increased in the plasma and brain after CA/CPR and that protein SNO levels in the brain decreased after 6 h in the placebo group, whereas GSNOR inhibitors, administered 15 min after ROSC, attenuated the upregulated GSNOR activity and restored protein SNO levels in the brain (90). Additionally, in wild-type mice after CA/CPR, GSNOR inhibitors improved the neurological deficit score and survival rate (81.8 vs. 36.4%, *P* = 0.031). Similarly, GSNOR-deleted mice prevented the reduction of the brain protein SNOs, suppressed neuronal damage, and improved survival. Both GSNOR inhibitor and GSNOR deletion attenuated the disruption of the BBB after CA/CPR. In PCAS patients, it was found that plasma GSNOR activity was higher than that in preoperative cardiac surgery patients or healthy volunteers (*P* < 0.0001) (90). In another publication, they demonstrated that plasma NO consumption in post-CA patients was 3-fold greater than in healthy volunteers (91). Overall, these observations suggest that increased GSNOR activity and the subsequent NO consumption may play an important pathogenetic role after ROSC and that the inhibition of GSNOR is a novel molecular target to improve neurological outcomes after CA/CPR (Figure 2).

Dezfulian et al. conducted a single-center, randomized, double-blind pilot clinical study to determine the effect of low-dose (~9.6 mg) intravenous sodium nitrate, a donor of NO, on OHCA patients (92). The patients were eligible to be enrolled in this study if the patient was successfully resuscitated from non-traumatic CA and survived to the intensive care unit (ICU) admission. Patients who had hypoxemia, hypotension, or inability to receive intravenous sodium nitrate within 12 h of onset were excluded. The results showed that there was no adverse effect on heart rate, systolic blood pressure, or blood methemoglobin level within 30 min of administration in the sodium nitrate group (*n* = 7) compared to the control group (*n* = 4). Plasma protein SNO and cGMP levels, which have protective effects on IRI (93), were elevated in the sodium nitrate group. The authors concluded that NO drug can be feasible for patients with PCAS and that further investigation is warranted (92). The same investigators are currently conducting a clinical trial to examine the effects of inhaled NO therapy on PCAS (ClinicalTrials.gov identifier: NCT04134078)^1^. Taken together, NO gas inhalation and NO-related drugs are currently one of the most promising pharmaceutical treatments for PCAS.

### Molecular Hydrogen (H_2_)

H_2_ is a colorless, odorless, and non-toxic gas at room temperature. H_2_ gas is explosive in air at a wide concentration range of 4.0–75.0% by volume, whereas in oxygen, the explosive limit is from 4.0 to 94.0% (94). The ignition point of H_2_ (527°C) is higher than that of gasoline (500°C), and it is difficult to ignite it spontaneously at standard conditions of pressure. These lines of evidence suggest that H_2_ is relatively safe in daily life when its concentration is <4% (94, 95). H_2_ is enzymatically metabolized as an energy source by providing electrons to METC. These enzymes catalyze the reversible redox reaction between H_2_ and its constituent two protons and two electrons (96). The use of inhaled H_2_ to diminish ischemic injury has been applied successfully in several rodent models, such as stroke (14, 97), acute myocardial infarction (MI) (98), and CA (60, 61). Consequently, clinical pilot studies have shown the beneficial effects of H_2_ in patients with acute MI (99) and OHCA (100).

**Figure 3:**
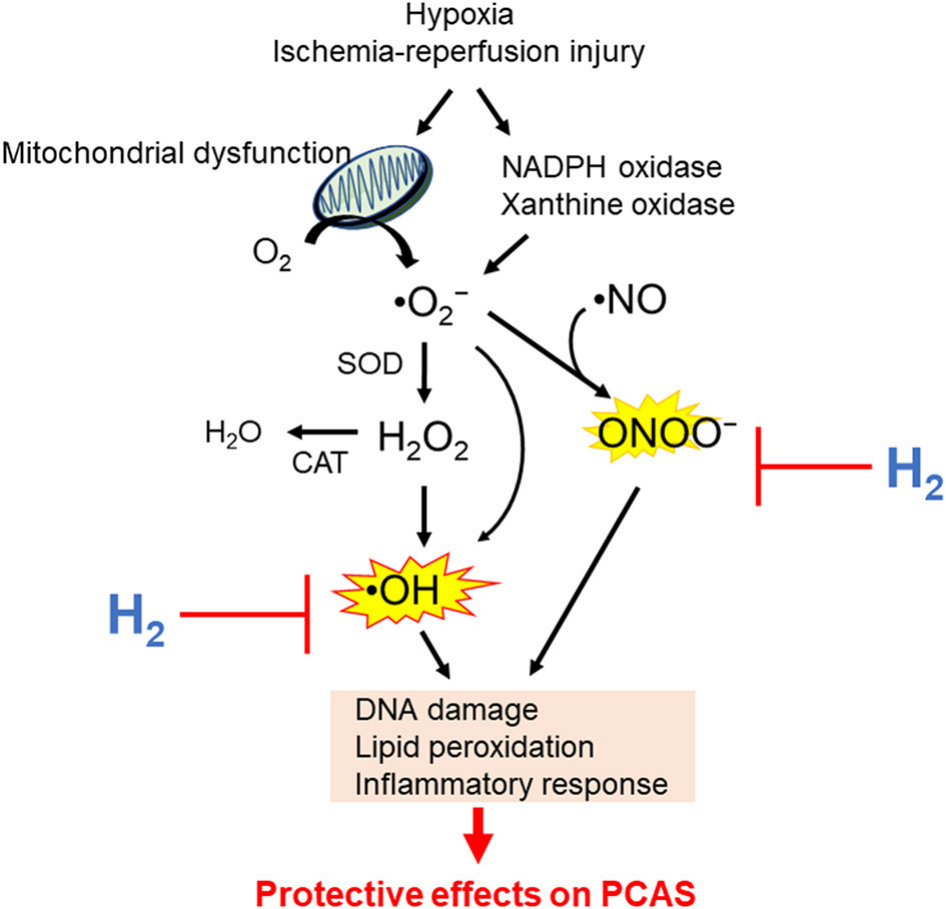
Potential mechanisms by which hydrogen (H_2_) inhalation improves outcomes in post–cardiac arrest syndrome (PCAS). NADPH, nicotinamide adenine dinucleotide phosphate; •${\text{O}}_{2^{-}}$, superoxide anion radicals; H_2_O_2_, hydrogen peroxide; •OH, hydroxyl radical; ONOO^−^, peroxynitrite; •NO, nitric oxide; SOD, superoxide dismutase; CAT, catalase.

While the mechanism of H_2_ protection has not been fully determined, many experts believe that its protective action is based on antioxidant properties with direct effects on ROS (101–104). Mitochondrial respiration chain, xanthine oxidase, uncoupling of NOS, and the family of nicotinamide adenine dinucleotide phosphate oxidases are significant sources of ROS (105). ROS includes superoxide anion radicals (•${\text{O}}_{2^{-}}$), hydrogen peroxide (H_2_O_2_), hydroxyl radical (•OH), peroxynitrite (ONOO^−^), and nitric oxide (NO•). •${\text{O}}_{2^{-}}$ is putatively the primary ROS mostly generated by electron leakage from the METC (106–109). H_2_O_2_ is enzymatically converted from •${\text{O}}_{2^{-}}$ by superoxide dismutase. •OH is a highly reactive, toxic ROS, and the major cause of oxidative stress (110); there is no detoxifying system for •OH *in vivo*. •OH is generated from H_2_O_2_ or •${\text{O}}_{2^{-}}$ through the Fenton or Weiss reaction in the presence of catalytically active metals such as Fe^2+^ and Cu^+^ (111). •${\text{O}}_{2^{-}}$ reacts with •NO to generate ONOO^−^, which is a highly active nitrogen species (112). Oxidative stress caused by H_2_O_2_ and •NO induces the production of enzymes involved in antioxidation and tolerance to protect the cells against oxidative stress, such as NF-E2–related factor 2 (113). Noteworthy, research has shown that many antioxidant supplements could not prevent cancer, MI, and atherosclerosis but rather, conversely, cause increased mortality (114–116); therefore, awareness of side effects is very important for developing an effective and safe antioxidant for ROS-related diseases. An ideal antioxidant should mitigate excessive oxidative stress without disturbing the redox homeostasis. In other words, an ideal molecule would simultaneously reduce strong oxidants such as •OH, while maintaining signaling molecules such as H_2_O_2_ (95). Preclinical studies have shown that H_2_ specifically quenches detrimental ROS such as •OH and ONOO^−^, while maintaining other less potent ROS (14). Potential mechanisms responsible for the beneficial effects of H_2_ on PACS are shown in Figure 3. However, more precise mechanisms of the beneficial effects of H_2_ remain elusive.

In 2007, Ohsawa et al. found that H_2_ acts as an antioxidant by selectively reducing highly cytotoxic ROS, such as •OH and ONOO^−^ in cultured cells, and that 2–4% of H_2_ has cytoprotective effects against IRI *in vivo* (14). Furthermore, it was demonstrated that H_2_ did not react with stable, lowly active ROS, such as H_2_O_2_, •${\text{O}}_{2^{-}}$, and •NO *in vitro* (14). In a rat model of MI, 2% H_2_ inhalation starting 5 min after the ligation of a coronary artery and continued for 60 min after reperfusion reduced the infarct size and inhibited the left ventricular remodeling (98). The authors confirmed that H_2_ diffuses into the myocardial ischemic tissues in a blood flow–independent manner, suggesting that H_2_ rapidly dissolved into the blood immediately after the start of inhalation and has the potential advantage of excellent diffusion even into ischemic regions (98). Another research group reported the inhibitory effect of H_2_ on myocardial IR damage in a dog model of acute MI (117). Moreover, the safety and efficacy of inhaled H_2_ for the prevention of reperfusion injury in patients with acute MI undergoing percutaneous coronary intervention have been assessed (99). In a single-center, open-label, pilot study, inhalation of 1.3% H_2_ did not reduce the infarct size during the acute phase after acute MI. However, the left ventricular stroke volumes assessed by magnetic resonance imaging (MRI) were improved at 6 months in comparison with 1 week after MI only in the H_2_ inhalation group (99). This suggests that H_2_ inhalation can be safely administered to patients with acute MI and can suppress adverse left ventricular remodeling at 6 months after infarction.

Hayashida et al. demonstrated that inhalation of 2% H_2_ starting at the beginning of CPR and administered for 2 h after ROSC significantly improves the outcomes in a rat model of CA with VF (60). H_2_ inhalation, but not TH, prevented an increase in the left ventricular end-diastolic pressure and myocardial injury and suppressed systemic inflammation after ROSC. The survival rate at 72 h after ROSC was 31% in the control group and 69% in both the TH and H_2_ groups and was even higher at 77% in the combined therapy (inhaled 2% H_2_ plus TH) group. Further, the same study group tested the benefit of H_2_ administered after ROSC under a normoxic condition, which was considered essential for clinical application (61). In this study, inhaled 1.3% H_2_ with 26% O2 was started 5 min after ROSC and continued for 2 h. The survival rates at 7 days were 38% in the control group, 71% in either the H_2_- or the TH-alone groups, and 86% in the combined therapy of H_2_ plus TH group. At 7 days after CA/CPR, H_2_ improved the motor activity and special memory assessed by the Y-maze test. Immunohistochemistry studies showed that H_2_ inhalation alone or in combination with TH inhibited neuronal injury in the hippocampus 7 days after ROSC. These results indicate that H_2_ inhalation after ROSC is as effective as TH for improving the neurological prognosis in rats with PCAS, whereas combined therapy had an additive effect (61). Further, Nemeth et al. showed that, in a hypoxic–ischemic encephalopathy piglet model, treatment with 2.1% H_2_ for 4 h reduced oxidative stress and improved neural recovery (118). Moreover, Cole et al. demonstrated the protective effects of inhaled H_2_ on neurologic injury after cardiopulmonary bypass in a porcine model of neonatal circulatory arrest (119).

In a single-center, prospective, open-label, single-arm study, Tamura et al. demonstrated the safety and feasibility of H_2_ inhalation after ROSC in comatose patients with a consciousness level ≤8 points on the Glasgow Coma Scale and a systolic blood pressure ≥90 mmHg (irrespective of vasopressor use) (100). In this study, the patients received 2% H_2_ for 18 h using a ventilator in combination with TTM of 33–36°C. The rates of survival with Cerebral Performance Category (CPC) 1–2 were assessed at 90 days after CA. The rates of survival with CPC 1–2 were assessed at 90 days after CA. One CA patient with severe pneumonia and septic shock died of respiratory deterioration 22 h after the discontinuation of H_2_ inhalation. An outcome of CPC 1 was achieved in 4 of all 5 eligible patients. The independent data monitoring committee concluded that no adverse event was attributable to inhaling hydrogen gas in this study. This study concluded that inhaled H_2_ could be feasible and performed safely in patients with PCAS. Currently, a phase II, multicenter, prospective, randomized, double-blind, placebo-controlled trial to verify the efficacy of H_2_ inhalation in patients with PCAS is underway (identifier: UMIN000019820) (120).

### Xenon

**Figure 4:**
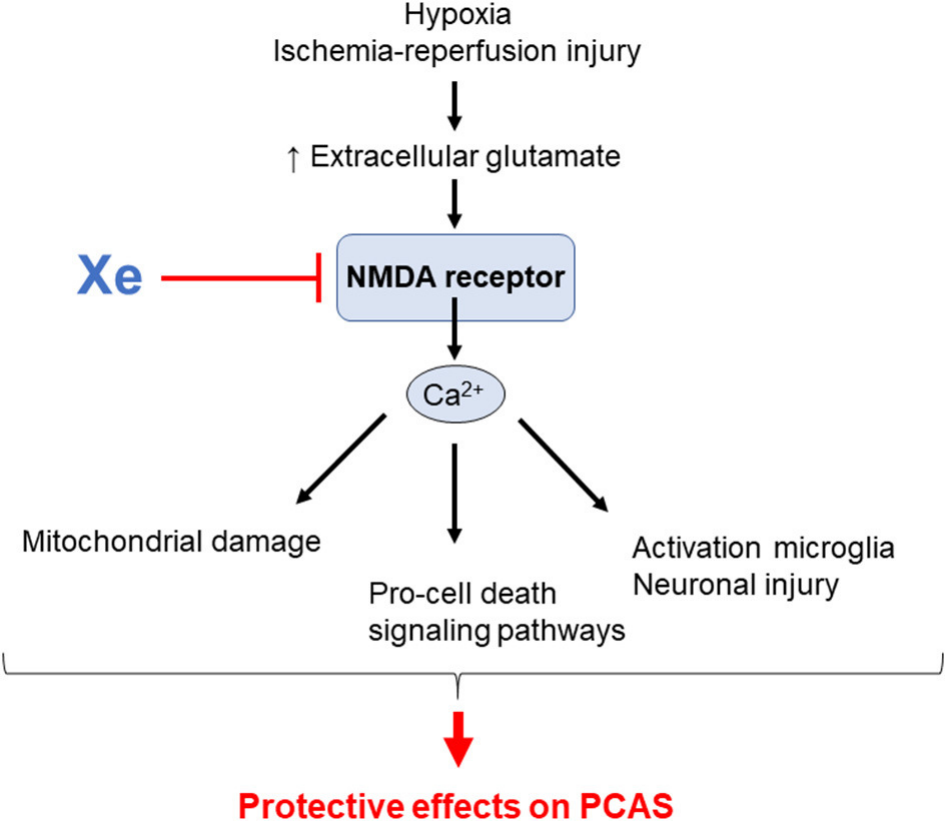
Potential mechanisms by which xenon (Xe) inhalation improves outcomes in post–cardiac arrest syndrome (PCAS). NMDA; *N*-methyl-d-aspartate.

Xe is one of the noble gases, which are the elements of group 18 on the periodic table. It has anesthetic properties, which were recognized ~50 years ago (121). It has the lowest blood–gas partition coefficient among anesthetic gases (122). It has the advantage of being non-flammable and non-teratogenic, and it has less cardiovascular effects and no adverse effects on cognitive function in animal models (123–126). In recent years, there has been increased interest in noble gases as novel treatments for ischemic and traumatic brain injury (127–129). Excessive activation of *N*-methyl-d-aspartate (NMDA)–type glutamate receptors is, in general, a key mechanism of excitotoxicity after brain injury (130, 131). During excitotoxicity, excessive glutamate release results in the activation of NMDA receptors, leading to calcium overload inside the neurons and the different types of neuroglia. This calcium overload triggers prodeath signaling pathways, ROS production, and mitochondrial damage (132–137), resulting in cell necrosis, apoptosis, and necroptosis (138). Additionally, the linkage of NMDA receptor and activation of microglia has been suggested (139, 140). Interestingly, studies have shown that NMDA-mediated excitotoxicity occurs unequally in different brain cells because neuroglia such as astrocytes do not express NMDA receptors in the same way as neurons do, making astrocytes relatively resistant to NMDA toxic effects (141, 142). Xe is an antagonist of NMDA-type glutamate receptors (143), and subsequent animal studies have reported that Xe has neuroprotective properties in animal models of stroke (144) and CA (59, 62, 145, 146). Additionally, Xe exhibits neuroprotection by inhibiting the activation of microglia and attenuating neural damage in the hippocampus after experimental subarachnoid hemorrhage (147). In a porcine model of CA, Fries et al. demonstrated that a single inhalation of Xe started 1 h after ROSC and continued for 1 h significantly improved functional recovery and reduced neuronal damage in a porcine model of CA (146). Furthermore, they showed that administration of Xe as early as 10 min after ROSC (59) and extending up to 5 h (146) did not result in additional neuroprotection. Subsequently, they demonstrated that only the combination of Xe and mild TH provided significant and persistent improvements in functional recovery in a clinically relevant, porcine model of CA/CPR. In contrast to mild TH alone, this approach also preserved cardiac output in the early postresuscitation period (62). Potential mechanisms responsible for the beneficial effects of Xe on the outcomes of PACS are shown in Figure 4.

In 2013, Arola et al. reported that Xe inhalation in combination with TH can be safely applied to patients with PCAS (148). Subsequently, Laitio et al. demonstrated that Xe had a neuroprotective effect on PCAS in a randomized, single-blind phase 2 clinical trial (149). In this study, 110 patients with PCAS admitted to the ICUs were randomly assigned to receive either TH alone (control group) or inhaled Xe in combination with TH (33°C) for 24 h (Xe group). The main inclusion criteria were the presence of a witness, initial electrocardiogram waveform VF or non-perfused ventricular tachycardia, and ROSC ≤45 min after resuscitation. The primary endpoint was the severity of ischemic white matter brain injury as evaluated by fractional anisotropy from diffusion tensor MRI, which was scheduled at 36–52 h after ROSC. Xe inhalation was started within 4 h after ROSC, and the mean end-tidal Xe concentration was 48.2%. The fractional anisotropy was significantly lower in 41.7% of the voxels in the control group than in the Xe group (i.e., 58.3% of the voxels did not significantly differ between the groups), indicating that cerebral white matter and myelin damage were suppressed in the Xe group. Specifically, the mean global fractional anisotropy value adjusted for age, sex, and site factors was 3.8% higher in the Xe group than in the control group (*P* = 0.006). The adjusted radial diffusivity value was 3.9% lower in the Xe group than in the control group (*P* = 0.03). There were no significant differences in the secondary endpoints of 6-month survival and brain function outcomes between the two groups (27.8% in the Xe group vs. 34.5% in the control group; adjusted hazard ratio, 0.49, *P* = 0.053) (149). Given that myelin is required for the normal functioning of the central nervous system and its damage is related to neurocognitive dysfunction (150), this study suggested that Xe may protect the cerebral white matter by preventing brain myelin injury after ROSC (149). Although there was no significant difference in survival in this study, Xe can be potentially a novel treatment for PCAS. Subsequently, Arola et al. demonstrated that among comatose survivors of OHCA, in comparison with TH alone, inhaled Xe combined with TH resulted in significantly reduced release of troponin-T, which suggests that Xe results in less severe myocardial injury, supporting its cardioprotective effects (151). These two recent clinical trials suggest the translational potential of Xe inhalation for the management of PCAS (149, 151). These studies have demonstrated that Xe inhalation in combination with TH is safe and feasible. Currently, phase III, multicenter, prospective, randomized, single-blind, placebo-controlled trial to evaluate the efficacy of Xe inhalation on neurofunctional outcomes after OHCA is underway (identifier: NCT03176186) ^2^.

Xe has many properties as an ideal general anesthetic, and because the noble gases emit light when an electric field is applied, they are often used as gas lasers in medical applications such as surgery (152). However, Xe has not been widely used in clinical practice as it is rare and relatively expensive (152). Therefore, as a relatively large amount of gas is expected to be used for inhalation therapy for PCAS, the feasibility in terms of the cost has been regarded as a potential problem. Hence, further investigations for the clinical application of Xe will be required.

### Other Considerations

NO is a toxic molecule (153) synthesized by NO synthases, which include three isoforms: neuronal NOS (NOS1), inducible NOS (NOS2), and endothelial NOS (NOS3) (154). In contrast to NO, mammalian cells do not have to produce intracellular Xe and H_2_. Although Xe is non-toxic, many of its compounds are toxic because of their strong oxidative properties. Xe readily penetrates the BBB, offering rapid onset of action, and titration of dose and response are rapid because of a low blood–gas partition coefficient (122). H_2_ has no known cytotoxicity even at high concentrations (155, 156).

As the primary target of NO, heme-based proteins play a central role. Integrated approaches revealed the physiological significance of NO on mitochondrial cytochrome c oxidase, a central mediator of mitochondrial respiration (157). Xe exerts neuroprotective effects by acting as an antagonist of the excitotoxic NMDA receptors (143). Excessive inflow of calcium mediated by NMDA receptors triggers complex biochemical cascades that ultimately lead to neuronal cell death (134). Although the molecular mechanisms of H_2_ have not yet been clarified, it has been shown that H_2_ does not reduce the oxidized heme of cytochrome c (14). In addition, a combined inhalation of NO plus H_2_ during IRI reduced the infarct size, maintained cardiac function, and reduced the generation of myocardial nitrotyrosine, which is associated with NO inhalation (158). Therefore, the primary targets of these gases seem to differ from different standpoints. Moreover, the optimal timing, concentration, and therapeutic window may differ among these gases. The exact underpinning mechanisms of these therapies remain to be unveiled in future studies. Elucidation of the mechanism of action will accelerate the translation into clinical. Summary of the past and current clinical trials investigating the effects of gases on PCAS are shown in Table 1. Because these gases are colorless, odorless, and difficult to check visually, they require a pressure regulator and flowmeter and must be handled under the local high-pressure gas safety control act, in clinical translation. It is important to clarify and establish the safety, toxicity, flammability, operability, and cost, individually, for the clinical application. However, we would like to emphasize that gas inhalation therapy may not require extensive equipment or advanced medical technology and is relatively easy to be introduced in a large number of facilities. In addition, dedicated gas cylinders can be installed in public areas or on ambulances to provide earlier therapeutic interventions during CPR or immediately after ROSC.

**Table 1:** Summary table of the past and current clinical trials on inhaled gases as therapies for PCAS (as of 1st, Dec, 2020).

**Intervention**	**Study title**	**Status**	**Locations**	**Identifier**
Nitric oxide	Improving outcomes in cardiac arrest with inhaled nitric oxide	Recruiting	USA	NCT04134078 ^1^
Molecular hydrogen	Efficacy of inhaled hydrogen on neurological outcome following brain ischemia during post-cardiac arrest care: HYBRID II trial (Phase II)	Recruiting	Japan	UMIN000019820 (120)
Xenon	Xenon for neuroprotection during post-cardiac arrest syndrome in comatose survivors of an out of hospital cardiac arrest (XePOHCAS)	Recruiting	USA	NCT03176186 ^2^
Molecular hydrogen	The effect and safety of hydrogen inhalation on outcome following brain ischemia during post cardiac arrest care: HYBRID study	Completed	Japan	UMIN000012381 (100)
Xenon	Effect of xenon and therapeutic hypothermia, on the brain and on neurological outcome following brain ischemia in cardiac arrest patients (Xe-hypotheca)	Completed	Finland	NCT00879892 (148, 149)
Nitric oxide	Inhaled nitric oxide after out-of-hospital cardiac arrest	Terminated^a^	USA	NCT03079102

a *The study has stopped early because of slow enrollment and planned change of institution by a principal investigator and will not start again*.

## Conclusion

We reviewed the developments in research on basic and clinical applications of NO, H_2_, and Xe for PCAS. The discussed studies provide insights on new frontiers regarding the fact that gas therapy may bring promising improvements in the prognosis of patients after ROSC. Nevertheless, there are substantial differences in the mechanisms by which these gases modulate IRI after ROSC. Further preclinical and clinical studies examining the combinations of standard post-CA care plus inhaled gas treatment to prevent IRI are warranted to improve outcomes in patients who are being failed by our current therapies.

## Author Contributions

KH: concept, design, and drafting manuscript. SM, KS, RT, TY, DR, RC, SG, EM, and LB: critical revision of the manuscript for important intellectual content. All authors have read and approved the manuscript. All authors contributed to the article and approved the submitted version.

## Conflict of Interest

The authors declare that the research was conducted in the absence of any commercial or financial relationships that could be construed as a potential conflict of interest.

## Footnotes

^1^https://clinicaltrials.gov/ct2/show/NCT04134078.

^2^https://clinicaltrials.gov/ct2/show/NCT03176186.
